# Toward a theory of STEM academic departments as racialized organizations

**DOI:** 10.3389/fsoc.2026.1907948

**Published:** 2026-09-25

**Authors:** Meaghan I. Pearson, Aireale J. Rodgers, Tatiane Russo-Tait

**Affiliations:** 1 Department of Engineering & Computer Science, University of Texas at Dallas, Dallas, TX, United States; 2 Department of Educational Leadership & Policy Analysis, University of Wisconsin-Madison, Madison, WI, United States; 3 Department of Cellular Biology, University of Georgia, Athens, Georgia

**Keywords:** academic departments, higher education, organization, racism, stem

## Abstract

Critical and STEM higher education scholars document how the fallacy of White supremacy has produced racialized macro-level (e.g., state) and micro-level (e.g., individual) structures gatekeeping the self-actualization and innovation of racially minoritized people and their communities. Although important, there has been an undertheorization of how meso-level structures like STEM academic departments gatekeep racially minoritized people and their knowledge systems. In this paper, we leverage theory of racialized organizations to illustrate how STEM academic departments in United States research-intensive, predominantly White institutions (PWIs) function as organizational sites of racial inequity. We use critical theory and empirical studies to explain how this racialized gatekeeping occurs through four structures: (1) graduate admissions and faculty hiring, (2) research, (3) instruction, and (4) service. Overall, this paper provides a conceptual and practical blueprint of how STEM academic departments, through their organizational rules and routines, maintain White racially hierarchies in STEM ecosystems. We conclude by offering examples of how STEM academic departments can engage, challenge, and disrupt their racially exclusionary organizational practices, so racially minoritized people and knowledge systems can survive and thrive in STEM ecosystems.

## 1 Introduction

Academic departments shape the development of science, technology, engineering, and mathematics (STEM) fields (Edwards, 1999; Ikenberry and Friedman, 1972). For example, STEM academic departments usher individuals into STEM disciplines by offering courses, conferring degrees, and hiring and promoting faculty (Ikenberry and Friedman, 1972). Additionally, STEM academic departments are home to cutting edge innovations that help advance society (Slaughter and Rhoades, 2004; Lawton-Smith, 2006). These traditional characterizations portray STEM academic departments as race-neutral organizational entities, in which all peoples and knowledge systems are validated and provided with the resources to thrive in STEM professions (Bess et al., 2023; Harding, 2006; Gonzales et al., 2018). Yet, research demonstrating that racially minoritized faculty and students experience unequal access, opportunities, and outcomes in STEM academic departments suggests otherwise (Blair-Loy and Cech, 2022; Flores et al., 2023; Perez et al., 2023).

The purpose of this paper is to present a theoretical argument for viewing STEM academic departments as racialized organizations—specifically within the context of United States (U.S.) research-intensive, predominantly White institutions (PWIs). According to Ray (2019), racialized organizations are those containing norms, policies, and practices that connect White cultural schemas to economic, material, social, and psychological resources. White cultural schemas are often unconscious, cognitive mental templates or default assumptions that associate White people’s norms, values, practices, knowledges, and culture as inherently superior to those of non-White peoples (Roberts and Rizzo, 2021). In racialized organizations, the connection between White cultural schemas to organizational resources produces White racial dominance in the organization and within society (Ray, 2019). We leverage Ray’s (2019) framework, supported by evidence from published empirical studies, to highlight the organizational mechanisms in STEM academic departments reproducing the privileging and legitimation of White people and their knowledge systems, while racially minoritized people and their knowledge systems are constrained. In other words, we unpack how STEM academic departments function in ways where White people and their knowledge production are provided with sufficient care and material support, while racially minoritized people and their knowledge production are gatekept from these supports and therefore harmed.

We, the authors, arrive at this work as educators and scholars who see ourselves as part of a collective effort to make STEM academic departments a place where racially minoritized people and their knowledge systems can thrive. Specifically, drawing inspiration from critical theory and critical race studies, our theorizing stems from the recognition that White Western societies continuously (re)produce structures embedded with racial and class mechanisms created to ensure racially minoritized groups remain a permanent subordinate class (Bonilla-Silva, 1997; Mills, 2019). This ubiquitous predicament is perpetuated in and through the work of STEM academic departments (Misra et al., 2024; Singh, 2026).

Theorizing how STEM academic departments in PWIs function as racialized organizations is important for several reasons. For starters, following decades of broadening participation efforts, the initiatives developed by leaders across STEM fields continuously fall short of their transformative potential (NCSES, 2023). Although important, such initiatives including bias workshop training, curricular transformations, and scholars’ research programs, often evade intervening on meso-level structures like STEM academic departments (Misra et al., 2024; Singh, 2026), leaving firmly intact the routines, policies, and procedures that maintain the existence of everyday racist encounters and disparities in our field (McGee, 2020). We propose that, by conceptualizing STEM academic departments as racialized organizations, we can expose how to challenge and disrupt how racialized beliefs, encounters, norms, and practices are arranged and entrenched in STEM higher education contexts.

Additionally, STEM academic departments are sites offering dramatic possibilities for racialized change efforts based on faculty autonomy and power within the higher education landscape (Chapman, 2020; Kezar et al., 2015). We recognize that STEM academic departments are not the sole organizational agents responsible for racial inequity in STEM fields and academia. Still, because of the centrality of STEM academic departments in the experiences and outcomes of faculty, staff, and students, we argue that there is a profound need to critically consider how to use their power to tangibly shift how their department can become a site of possibility and progress (Reinholz and Apkarian, 2018).^1^

This paper is organized as follows: first, we introduce Ray’s (2019) theory of racialized organizations. Then, we offer a description of STEM academic departments and their organizational routines and structures. Next, we explain how each of the structures in STEM academic departments contain racially exclusionary mechanisms that work to restrict agency and resources from racially minoritized people, while amplifying the agency and resources of White people. Later, we explain how this unequal distribution of resources has broader implications for societal structures and individual people. We conclude the paper by discussing the theoretical implications for research and offering suggestions for how organizational actors in STEM academic departments can mitigate the mechanisms that perpetuate racial stratification and better support racially minoritized populations.

## 2 Theoretical foundations: theory of racialized organizations

For decades, mainstream scholarship on race and racism has documented how racial ideologies influence institutional and individual relations (Fanon, 1986; Golash-Boza, 2016; Mills, 2019; Omi and Winant, 1994; Salter and Adams, 2013; Steele and Aronson, 1995). Racial ideologies are systems of beliefs and values affirming one racial group (e.g., White) as superior to other racial groups (e.g., non-White; Golash-Boza, 2016; Bonilla-Silva, 1997). At the institutional level, scholars demonstrate how the government and the economy act as racial institutions (i.e., those macro-level structures developed to facilitate the access and allocation of agency and resources to White populations) by ostensibly and ironically designing “color-blind” laws and practices that nevertheless hinders the economic and social stability of racially minoritized populations (Alexander, 2010; Bonilla-Silva, 1997; Kilty and de Haymes, 2012; Roberts, 2014). With a gaze toward the innerworkings of institutions, racism scholarship illustrates how institutions across U.S. society socialize and encourage individuals to embrace ideologies that provide unearned advantages to White racial groups (Allport, 1954; Bigler and Liben, 2006; Bonilla-Silva, 2007; Roberts and Rizzo, 2021).

More recently, Ray (2019) advocated for a deeper understanding of how organizations influence racialized power relations. Prior work has depicted organizations as groups of people who work together to produce a common goal (Scott and Davis, 2001). Within organizational theory, scholars, known as institutionalists, have highlighted how organizational goals and procedures are a byproduct of their external environment (Scott, 2005). For example, universities were forced to alter their race-conscious admissions policies following the Supreme Court ruling in the *Students for Fair Admissions, Inc v. President and Fellows of Harvard College* lawsuit (Roman, 2023). Institutionalists argue organizational actions are not merely rational or objective; rather, they propose organizational behavior is influenced by exogenous shocks that jolt institutions to change (DiMaggio and Powell, 1983; Meyer and Rowan, 1977). Thus, the primary purpose of the institution—actualized through organizations—is self-maintenance. However, institutionalists mostly examine environmental systems like the economy or the government (Scott, 2005; Thornton and Ocasio, 2008), usually separately from racialized social systems (Bonilla-Silva, 1997).

Ray (2019) argues that analyses of racial stratification focused on the institutional (e.g., government) and individual (e.g., microaggressions) levels hide the role that organizations, located at the meso-level, play in maintaining racial stratification. Thus, a contribution of Ray’s (2019) work is that it goes beyond a broad argument insisting that organizations are racialized and shifts toward animating how they (re)produce racialization. Ray (2019) argues racialized organizations leverage White cultural schemas that shape organizational norms, processes, and resource allocation (Ray, 2019). Within these organizations, the unequal distribution of resources and agency between White people and racially minoritized people creates White racial hierarchies (Ray, 2019). Consequently, White racial hierarchies help to validate racial ideologies asserting the superiority of Whiteness within and outside of their organizational context (Ray, 2019). Ray (2019) posits racialized organizations maintain White racial hierarchies in four primary ways: (1) constraining people of color’s agency, (2) disadvantaging people of color’s access to resources and networks, (3) maintaining the superiority of Whiteness in credentials, and (4) claiming, but not practicing a commitment to dismantling racism (i.e., decoupling).

Overall, Ray’s (2019) theory of racialized organizations positions racism as a multi-level phenomenon. At the institutional level, racial institutions like governmental bodies create state-sanctioned structures (e.g., laws and categories) that disproportionally allocate state resources, membership, and inclusion across racial groups (Ray, 2019). At the organizational level, racialized organizations uphold institutional and individual racial exclusion by disproportionally allocating organizational resources and agency across racial groups (Ray, 2019). At the individual level, individuals leverage racial ideologies to justify the disproportionate allocation of institutional and organizational resources across racial groups (Ray, 2019). Thus, institutions—through their propensity to legitimize and uphold the White racial order—support the everyday internalization and embodiment of racial ideologies at the individual and interpersonal levels (Bonilla-Silva, 1997). To understand and eventually disrupt this, however, Ray (2019) emphasizes the importance of interrogating the organizational routines and policies complicit in perpetuating this process.

Ray’s (2019) framework expands on existing scholarship theorizing how White supremacy functions in higher education. Specifically, higher education scholars have used institutional Whiteness and epistemic oppression as conceptual frames to document how universities and colleges contribute to the White racial order (Cabrera et al., 2017; Moore, 2008; Settles et al., 2024). First, institutional Whiteness scholarship situates universities and colleges as spaces where Whiteness is reflected in campus ecologies, climates, curricula, and social interactions (Duran et al., 2022; Joseph-Salisbury, 2019; Scott et al., 2024). Although important, institutional Whiteness as a theoretical frame often does not capture variations between broader institutional (e.g., university or college level) and department level mechanisms (Ray, 2019; Southern, 2025). Additionally, epistemic oppression scholarship illustrates how academic contexts leverage Whiteness to assess scholarly contributions, particularly in departmental faculty evaluations (Settles et al., 2019, 2024). While valuable, epistemic oppression scholarship’s theoretical focus on the research level does not include how additional structures like instruction operate within STEM academic departments. Thus, we argue Ray’s (2019) framework offers the ability to account for multiple structural processes occurring at the STEM academic department level.

Recently, education scholars have increasingly utilized the theory of racialized organizations to emphasize how organizations’ patterns of resource allocation serve as engines of racial inequity (Perez et al., 2023; Poon et al., 2024). For example, Nguemeni Tiako et al. (2022) argued for characterizing medical schools as racialized organizations based on their racially exclusionary admissions policies, evaluations, and climates. McCambly et al. (2023) explained how U.S. community colleges are a type of racialized organization that “are often as marginalized as the students they serve” given the systematic underfunding of these institutions (p. 658). Additionally, Vega et al. (2022) bridged racialized organizations with the concept of intersectional consciousness to demonstrate how “racialized ideologies designed to whiten organizations and further invisibilize Black and Indigenous Latinx people” operate within Hispanic Serving Institutions (p. 3). Ultimately, this growing body of scholarship provides evidence detailing how racial power dynamics shape how colleges and universities unevenly distribute resources between White and racially minoritized people. This uneven distribution across colleges and universities extends to the broader ecosystem in ways that reproduce White racial hierarchies within and across academic departments and higher education institutions (Dancy et al., 2018; Patton, 2016).

We propose that Ray’s (2019) theory of racialized organizations offers insight into how STEM academic departments are organized around White cultural schemas and ideologies that lead to resource allocation that advantages White people and reproduces racial inequities. For example, Nakajima et al. (2022) highlighted how computer science departments strayed away from developing racial equity supports, causing students of color to navigate negative racial climates. By leveraging Ray’s (2019) theory of racialized organization, Nakajima et al. (2022) demonstrated how the organizational routines and practices in STEM academic departments maintain White power and privilege dynamics. Thus, by extending Ray’s (2019) theory of racialized organizations to STEM academic departments, we build on the work of Nakajima et al. (2022) and others by offering a conceptual template of how existing racial inequities emerge from the relationship these departments have with White racial ideologies and schemas (McGee, 2020; Nakajima et al., 2022). In the following section, we briefly turn our attention to literature highlighting background information on the structures, processes, and functions of STEM academic departments.

## 3 An overview of STEM academic department structures and processes

STEM academic departments are organizational units dedicated to advancing the knowledge production of a given STEM discipline, while also contributing to the university and college environment in which they are embedded (Ikenberry and Friedman, 1972). Historically, early colleges and universities utilized an apprenticeship model to promote student learning, where individual professors educated students across multiple disciplinary fields (Anderson, 1976). As the global demand for higher education grew in the 19th century, colleges and universities restructured themselves to accommodate student demand and the rise of new disciplinary fields (Seagren et al., 1993). Consequently, academic departments were constructed to centralize a discipline’s knowledge production in one organizational unit within colleges and universities (Seagren et al., 1993). Currently, STEM academic departments make important contributions that advance the status of both their discipline and institution through several structures and processes, including providing disciplinary training and support (e.g., instruction, service), producing innovative scholarly contributions (e.g., research), and awarding disciplinary credentials (e.g., granting degrees, faculty hiring and promotion; Ikenberry and Friedman, 1972).

People have many different roles in STEM academic departments. First, undergraduate students are considered novices and generally have the least positional power in STEM academic departments. Undergraduate students gain expertise by successfully passing courses within their department. Completing coursework enables students to earn an undergraduate degree, signaling their status as a STEM practitioner. Next, graduate students hold a mid-level role in STEM academic departments. As master’s and doctoral students, graduate students are expected to receive additional training through upper-level coursework and activities that demonstrate their ability to apply curricula and content knowledge to real-world contexts (e.g., internships, research, and teaching assistantships). Some people describe this shift as moving from being a consumer toward becoming a producer of knowledge in the discipline (Wenger, 1999). As such, a graduate degree confers legitimacy as a disciplinary expert with innovative scholarly contributions and specialized practice (Liera et al., 2023).

Next, faculty members hold the highest positions in STEM academic departments. Faculty members have credentials (i.e., graduate degrees) that legitimize their disciplinary expertise within the department, college, university, and academic field. With this expertise, faculty members are given primary responsibility over curricular and instructional decisions, degree requirements, hiring and promotion of other faculty members, and graduate student admissions. Faculty members are classified as adjunct or contingent faculty and tenured/tenure-track faculty. While adjunct faculty are known to make important contributions to teaching and learning in academic departments (Kezar et al., 2019), we set our gaze on tenured and tenure-track faculty because they traditionally have the decision-making power in academic departments. Tenured or tenure-track faculty members hold positions across multiple levels including assistant, associate, and full professor. Tenured or tenure-track faculty members who hold the highest ranks have more power in departmental decision-making and operating procedures. Additionally, each academic department has a department chair who is a tenured faculty member and is at the top of the organizational ladder. Department chairs have primary responsibility for overseeing all departmental operations, like governance, promotion and hiring, student affairs, and budget decision-making, etc.

Other significant members of STEM academic departments include administrative staff and postdoctoral researchers. Administrative staff assist departmental leadership’s efforts for student affairs, curriculum, hiring and promotion policies, and research structure. Postdoctoral researchers support the research enterprise and productivity of faculty in academic departments. Although administrative staff and postdoctoral roles play pivotal roles in STEM academic departments, this paper focuses on the experiences of students and faculty based on their extensive coverage in STEM higher education research.

Following Ray (2019), our theoretical framework proposes STEM academic departments in research intensive PWIs contain four key structures constraining the agency of faculty and students of color. This framework attempts to illuminate the mundane parts of the organization of STEM academic departments where rules and routines shape access to resources. Rules are formal or informal standards of how individuals are expected to behave in organizations. Routines are formal or informal practices assisting how organizations achieve their goals. According to Ray (2019), structures become racialized when the association between rules, routines, and resources are influenced by White cultural schemas. That is, organizational rules and routines operate through cultural Whiteness, shaping who belongs or is a “fit” by defining competence through aesthetic and interactional norms before a formal resource allocation decision is made (e.g., hiring or admissions). For this paper, we propose that STEM academic departments are composed of four racialized structures: (1) graduate admissions and hiring, (2) research, (3) instruction, and (4) service (refer to Figure 1 for an overview of this process). In our framework, we leverage and integrate Ray’s (2019) four mechanisms—agency constraints, unequal resources, Whiteness as a credential, racialized decoupling—to demonstrate how STEM academic departments engage in racialized processes that contribute to an unequal distribution across racial groups. Consequently, our theoretical framework argues the unequal distribution of resources maintains the White racialized structure of STEM academic departments.

**Figure 1:**
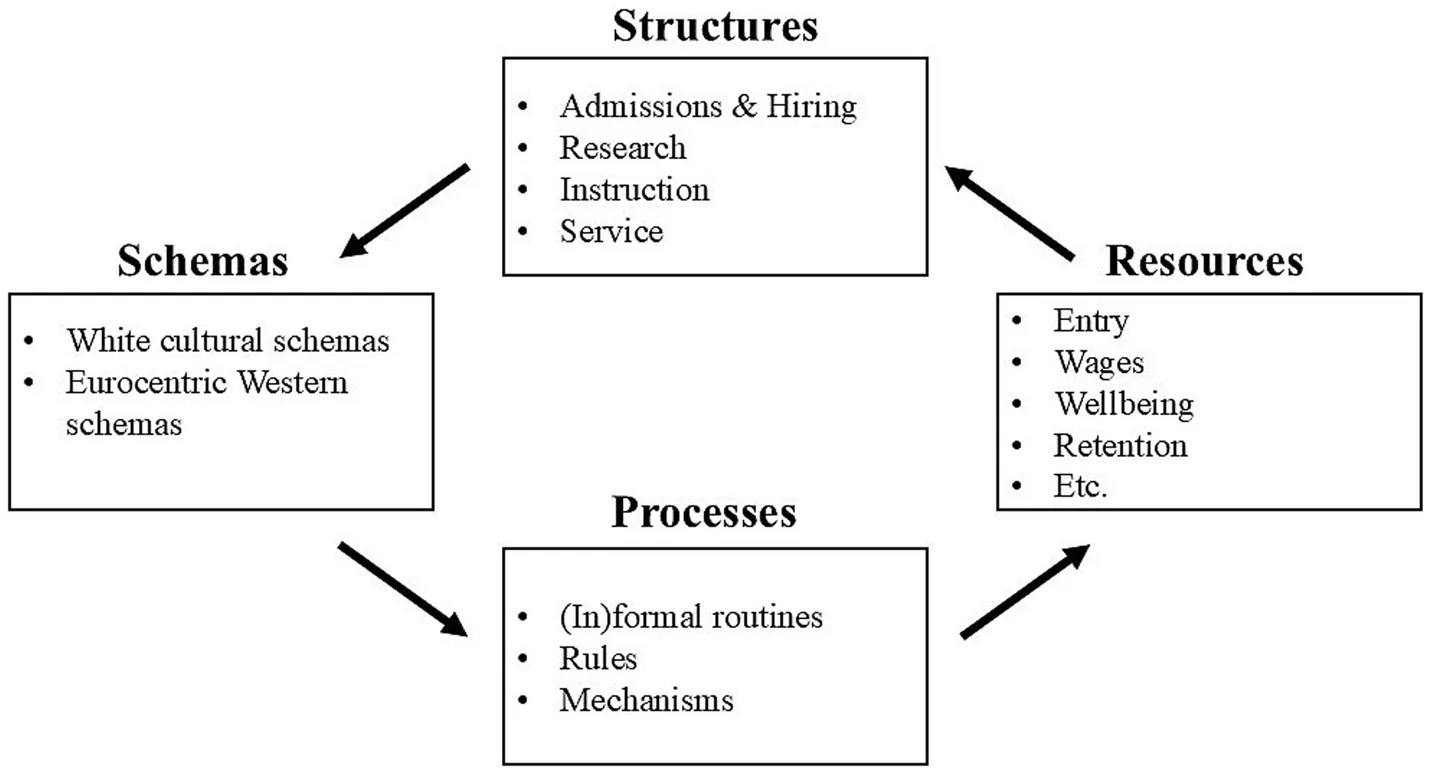
The racialized organizational rules, routines, and resource flows in STEM academic departments.

## 4 Theorizing how organizational structures in STEM academic departments reproduce racialized organizations

In this section, we present a theoretical framework explaining how STEM academic departments in predominately White colleges and universities function as racialized organizations. First, we provide a brief description of each structure. Then, relying on Ray’s (2019) theory of racialized organizations, we explain how although appearing neutral on the surface, each structure involves racialized organizational mechanisms that work to constrain the economic, material, and psychological resources of racially minoritized peoples and their knowledge systems, while leaving largely untroubled the fallacy of White supremacy. We acknowledge the organizational rules and routines of STEM academic departments in PWIs are not always separate from the influence of external forces like universities, disciplines, and funding agencies. However, we argue that although influenced by external forces, STEM academic departments make decisions contributing to the subordination of faculty and students of color, making them an active role player in racial inequity. Lastly, we conclude each section by illustrating how the racialized organization of each structure has broader racialized implications. For a summary of our arguments, refer to Table 1.

**Table 1:** Overview of how STEM academic departments function as racialized organizations.

Structure	Mechanism	Formal and informal rules and routines	Consequence to faculty of color	Consequence to students of color
Graduate admissions and faculty hiring	The (re)production of racial hierarchies through racialized conceptions of merit and legitimacy, transforming Whiteness into a credential.	Informal rules use color-evasive language like “fit,” becoming a racial filter where White candidates are given the benefit of the doubt, and candidates of color are unduly scrutinized.	Faculty of color on the tenure-track need to overperform to be assumed legitimate.	Graduate students of color face similar scrutiny as well as chilly racial climates and isolation.
Research	Epistemic oppression upholds White Western knowledge systems while devaluing minoritized knowledge systems.	“Good” science is connected to objective, ordered, and rational ways of knowing (White Western norms), minimizing scientific contributions focused on social applications or community-based approaches often pursued by scholars of color.	Faculty of color experience a devaluation of their scholarship by academic departments and national agencies, which constrains their access to psychological and material resources (e.g., personnel, lab space) needed for tenure and promotion.	Students of color face racialized and classed disparities in accessing research opportunities, which are critical for advancing their STEM careers.
Instruction	The structure is organized to gatekeep who can pass courses.	Curriculum prioritizes White western ways of knowing and lived experiences which advantages White students and fails to account for racial and classed inequities in K-12 schooling.	Faculty of color with heavy teaching loads have less time for research and self-care, negatively impacting their wellbeing and job security.	Students of color experience psychological stressors like stereotype threat and instruction not reflective of their lived experiences.
Service	Racialized decoupling minimizes the importance of racial equity work in formal evaluation metrics.	Faculty and students of color are informally expected to take on “invisible labor,” such as unpaid mentorship for students of color.	Faculty of color experience “cultural taxation,” performing time-consuming and emotionally draining service without compensation or reduced teaching/research loads.	Students of color also perform racial equity labor and have less time for coursework, research, and supporting their wellbeing.

Ostensibly neutral organizational practices in STEM academic departments in PWIs maintain racial stratification by disproportionately allocating resources and agency across racial groups, guided by dominant White western cultural schemas. These formal and informal organizational rules and routines legitimize unequal outcomes through seemingly race-neutral processes.

### 4.1 Structure 1: graduate admissions and faculty hiring

The first structure we interrogate is graduate admissions and faculty hiring. As we described, faculty members and graduate students are at the upper levels of the organizational hierarchy in STEM academic departments. Faculty members are at the top of the organizational ladder based on their control over decision-making on hiring and admissions processes, tenure and promotion, curricular requirements, and institutional service. At the next organizational tier, graduate students are responsible for supporting research and instruction in the department.

STEM academic departments have organizational rules and routines through which they must make decisions to ensure the department is appropriately staffed with graduate students and faculty members who can support departmental goals. The organizational decision-making surrounding how STEM academic departments grant access to who becomes a faculty member and graduate student has broader implications for the organizational arrangements of STEM academic departments. In this section, we describe how although graduate admissions and faculty hiring appear neutral, these organizational decisions often operate in ways where White individuals experience advantages in these positions.

#### 4.1.1 Racialized mechanism of graduate admissions and faculty hiring

As racialized organizations, STEM academic departments are sites of the (re)production of racial hierarchies. Academic departments are contexts where “racialized conceptions of merit and legitimacy are reinforced by the social contexts and organizational cultures within which evaluation and decision-making occur” (Posselt et al., 2020, p. 21). These racialized conceptions involve the ways that speech, dress, attitude, and professionalism are coded through a White cultural frame. In STEM academic departments, the prioritization of objective and neutral values obscures the racialized dynamics in faculty hiring and graduate admissions (Blair-Loy and Cech, 2022; Posselt, 2016). Formal and informal rules regarding evaluations and decisions for admissions and hiring prioritize credentials, networks, status, and forms of knowledge that align with White, affluent, cis hetero male, and Western norms (Blair-Loy and Cech, 2022; Eaton et al., 2020; Settles et al., 2021). While STEM academic departments may appear meritocratic, they often use Whiteness as the unmarked standard by which faculty and graduate student candidates are judged (Blair-Loy and Cech, 2022).

STEM academic departments construct formal metrics and processes of how faculty and students are granted entry. Formal processes, even ostensibly race-neutral rubrics, tend to favor candidates who conform to White-man centric norms in STEM academic departments (Eaton et al., 2020; Settles et al., 2024). In faculty hiring, STEM academic departments contain formal rules where prospective candidates are assessed on criteria like receiving a doctoral degree from prestigious institutions (which are often predominantly White), being in well-funded labs with well-connected mentors, having many publications in high impact, elite journals, and success in acquiring fellowships, and other extramural funding (Blair-Loy and Cech, 2022; Clauset et al., 2015; Posselt, 2016). Racially minoritized candidates are less likely to have access to these resources due to systemic underinvestment and racialized structural inequities in minority-serving educational institutions (Cunningham et al., 2014; Douglas-Gabriel, 2023; Goslee, 2023; National Academies of Sciences, Engineering, and Medicine [NASEM], 2023).

In the formal hiring process, STEM academic departments’ assessments of a candidates’ scholarly potential reflect White Western centric norms (Blair-Loy and Cech, 2022; Liera and Hernandez, 2021). STEM academic departments engage in processes of epistemic oppression in which their hiring organizational processes devalue the types of knowledge production that scholars of color tend to be interested in (Harding, 2006; Gonzales et al., 2025; Masters-Waage et al., 2024; Settles et al., 2019). For example, STEM academic departments often devalue work focusing on specific populations, exploring disparities and social justice issues, and community-engaged research (Gonzales et al., 2025; Masters-Waage et al., 2024; Settles et al., 2019). Scientists often resist considering such scholarship as rigorous, objective, and legitimate, which not only affects hiring and admissions, but also grant funding decisions (Hoppe et al., 2019; Ginther et al., 2011; Taffe and Gilpin, 2021). In these ways, the standardized metrics used by STEM academic departments to guide decisions about faculty hiring and promotion hinder scholars of color whose scholarship often challenges mainstream STEM paradigms (e.g., basic versus applied research, emphasis on interdisciplinarity) and centers community-engaged research (McCambly et al., 2025; Masters-Waage et al., 2024).

Similarly, the formal rules with governing graduate admissions in STEM academic departments appear race neutral, but also favor candidates who adhere to White-centric norms (Posselt, 2016). Although informed by university and disciplinary expectations, academic departments have the power to consider factors that may affect a prospective candidate’s portfolio (McCambly et al., 2025; Posselt, 2014). In graduate admissions, STEM academic departments value metrics like GPA and standardized test scores, letters of recommendation, personal statements, and a history of prior research experiences. STEM academic departments’ reliance on standardized test scores like the Graduate Record Exam (GRE) for admissions decisions ignores research findings demonstrating how these measures a) fail to accurately predict student performance in graduate school, and b) are known to disadvantage students of color (Feldon et al., 2024; Posselt et al., 2019).

STEM academic departments also constrain the agency of faculty and students of color through informal gatekeeping, biases, and color-evasive language such as “fit.” Here, “fit” acts as a cultural filter that scrutinizes minoritized scholars’ belonging while naturalizing White ways of being as the unmarked standard. For example, informal rules in search committee deliberations often reflect committee members’ implicit biases in which considerations of the “top candidate” are often linked with Whiteness (Liera and Hernandez, 2021; Posselt et al., 2025). White faculty members often favor prospective candidates who are culturally similar or recommended by known elite networks, which are often predominantly White based on the composition of most STEM academic departments (Kehal, 2022; Posselt, 2016). Additionally, White candidates are often assumed to be competent and seen for their potential as future leaders and innovators, and any gaps in their record are given the benefit of the doubt (Correll and Ridgeway, 2003; Gonzales et al., 2025). Meanwhile, candidates of color must overperform to be assumed as legitimate, they are not judged on potential, and every gap in their record is unduly scrutinized by the hiring committee (Blair-Loy and Cech, 2022; Correll and Ridgeway, 2003; Liera and Hernandez, 2021).

#### 4.1.2 Resource constraints in graduate admissions and faculty hiring

Formal and informal routines of privileging White candidates in faculty hiring and graduate school decision-making create an unequal distribution of resources (e.g., material, social, psychological, and ideological). STEM academic departments often hire faculty of color as contingent labor in non-tenure track or contingent faculty positions (Finkelstein et al., 2017). Non-tenure track faculty are disproportionately women and people of color PhD holders who are as equally qualified for research positions as tenure-track faculty, but who instead are hired to take on positions with less recognition, higher teaching loads, low pay, and no job security (Baker and Koedel, 2024; Finkelstein et al., 2017; O'Meara et al., 2018). Consequently, academic departments are more likely to constrain the agency of non-tenure track faculty of color through heavy teaching loads in high enrollment courses, lack of resources and support, as well as curricular expectations to “cover” an inordinate amount of content (Benjamin, 2000; Jolley et al., 2014). Further, their location within the academic departments’ organizational structure often means that they are not given the power to participate in faculty governance and contribute to changes in policies and practices of the department or institution (Forsythe and Hsu, 2025). Thus, we argue that STEM academic departments can amplify existing racial inequities by not properly considering and contextualizing the factors influencing the scholarly contributions developed by faculty of color. In return, STEM academic departments reinforce broader racial disparities in institutions and innovation.

In STEM academic departments, graduate students also experience unequal resource distribution along racial lines. STEM academic departments produce racially chilly climates for graduate students of color (Robinson et al., 2016). The minimal racial diversity in these departments causes feelings of isolation and lack of belonging for graduate students of color (Rodriguez et al., 2022). Additionally, STEM academic departments’ failure to transform their racialized structure means graduate students of color must exert large amounts of their time and energy participating in diversity work (Perez et al., 2023). Therefore, graduate students of color who engage in racial equity labor may have less time to do research, hindering their future research career aspirations (Perez et al., 2022).

#### 4.1.3 Implications of racialized organizational rule in graduate admissions and faculty hiring

Overall, STEM academic departments function as racialized organizations by maintaining White racial hierarchies through their graduate admissions and faculty hiring decisions. STEM academic departments use Whiteness as a credential to determine how faculty and graduate students gain their role in departments. In return, the credentialing of Whiteness through routine behavior and rules perpetuates an underrepresentation of faculty and graduate students of color in STEM academic departments. Without diverse mentors and gatekeepers, those who make the decisions continue to value Whiteness and the cycle of exclusion continues. Based on these actions, STEM academic departments constrain the agency of students and faculty of color by hindering their career mobility, emotional, and physical wellbeing. Thus, the legitimization of unequal racial outcomes occurs through seemingly neutral routines, which ultimately shapes who gets into STEM academic departments and who does not, reinforcing the White status quo.

### 4.2 Structure 2: research

We now turn to the second structure in STEM academic departments: research. In research-based universities and colleges, STEM academic departments have organizational expectations for research production. Research conducted in STEM academic departments is expected to advance STEM academic disciplines, departments, and universities. At the disciplinary level, research production builds new knowledge for the discipline utilizing disciplinary traditions and conventions. At the department and university level, research production should offer social and economic benefits to the department and university system. Based on these organizational expectations, STEM academic departments have organizational rules for the research production process. First, faculty members are expected to lead research production in the department. Second, students at undergraduate and graduate levels have formal and informal expectations to engage in research to be properly socialized into the discipline.

Within STEM academic departments, the organizational expectation for research operates in racially exclusionary ways. STEM academic departments develop organizational mechanisms that enact epistemic oppressive practices (Dotson, 2014; Harding, 2006; Settles et al., 2024). Epistemic oppression refers to systemic acts of upholding White Western knowledge producers and White Western knowledge systems, while simultaneously devaluing knowers from minoritized social groups and their knowledge systems (Dotson, 2014; Harding, 2006). In STEM academic departments, epistemic oppression operates through the department constraining who can produce research and which type of research is valued (Settles et al., 2021, 2024).

In this section, we describe two ways of how research production in STEM academic departments is racially exclusionary for faculty and students of color. First, we explain how STEM academic departments are organized to ensure faculty of color experience different forms of exclusion in their research endeavors. Second, we outline how STEM academic departments are organizationally arranged to hinder how undergraduate and graduate students of color can produce research and see themselves as knowledge producers. Together, we argue these exclusionary organizational mechanisms ensure White Western knowers and knowledge are strengthened, while racially minoritized knowers and knowledge are constrained in STEM academic departments.

#### 4.2.1 Racialized mechanism in research for faculty of color

In academic departments, departmental assessments to evaluate a faculty member’s scholarly contribution rely on metrics favoring White populations (Settles et al., 2024). STEM academic departments set up the expectations and make decisions to evaluate faculty based on the amount of grant funding they receive from external and internal agencies. These grant funding metrics fail to account for how funding agencies are less likely to award funding to proposals by scholars of color even when accounting for training and submission attempts (Hoppe et al., 2019). Additionally, STEM academic departments make decisions to evaluate faculty’s research based on its perceived impact, typically assessed by h-index indicators. Across academic disciplines, researchers from minoritized social groups are more likely to produce new conceptual insights (Hofstra et al., 2020). However, these insights are less likely to be taken up by their fields of study in comparison to the insights produced by White scholars (Hofstra et al., 2020). Therefore, STEM academic departments’ use of citational indicators to assess a scholar’s impact may disregard how citational metrics reflect White racial biases that undermine innovative contributions by scholars of color and affect such scholar’s persistence in the department.

Another way STEM departmental research evaluation centers White Western norms is through the assessment of ‘good’ scientific work. In STEM academic departments, White Western ways of knowing reign supreme in which ‘good’ science is connected to objective, ordered, and rational ways of knowing (Harding, 2006; Blair-Loy and Cech, 2022). Academic departmental assessments often minimize the scientific contributions of faculty of color whose scientific work centers social applications (Gonzales et al., 2025; Settles et al., 2021, 2024). This minimization aligns with scholarship on how the National Institutes of Health (NIH) is less likely to fund scholarship with community-based approaches in comparison to research with mechanistic applications (Hoppe et al., 2019). According to this scholarship, researchers of color were more likely to submit NIH research proposals on intervention or community topics whereas White researchers were more likely to submit NIH research proposals with mechanistic topics, creating racial differences in NIH funding allocation (Hoppe et al., 2019). This scholarship aligns with critical and feminist scholars explaining how researchers from minority groups view scientific work as a vehicle for addressing social and community applications, abandoning epistemic beliefs of scientific research being disconnected from the world and those who inhabit it (Harding, 2006; McGee and Bentley, 2017; McGee, 2021).

Additionally, informal processes regarding ‘fit’ in STEM academic departments shape how faculty of color’s research is evaluated. Although tenure and promotion decisions are connected to a faculty member’s research, teaching, and service, departmental perception regarding a faculty member’s perceived ‘fit’ in the department matters as well (White-Lewis, 2020). As mentioned earlier, departmental assessments of scholarly impact privilege White faculty due to societal schemas of centering White Western ways of knowing and being (Settles et al., 2021). In STEM academic departments, departmental members and climates often fail to recognize the intellectual contributions, grant funding allocation, and national recognition of faculty of color (Blair-Loy and Cech, 2022; Settles et al., 2021, 2024). For example, university promotion and tenure committees have been found more likely to reject the promotion of faculty of color in comparison to White faculty with similar below-average h-index scores, suggesting variations in how scholarly productivity is evaluated across racial groups (Masters-Waage et al., 2024). This finding coincides with scholarship illustrating how STEM faculty of color explain not being evaluated solely for their research production, but also being penalized for insubstantial standards like ‘not being seen enough in the department’ in their promotion evaluations (Jackson et al., 2023).

#### 4.2.2 Resource constraints in research for faculty of color

Racially exclusionary departmental research assessments constrain the material resources of faculty of color in STEM academic departments. The underfunding of faculty of color’s research by national funding agencies ensures faculty of color have less access to resources to hire personnel (e.g., graduate students, lab managers) and physical support (e.g., lab space) needed to produce high-quality research (Ginther et al., 2011; Chen et al., 2022). The unequal access of resources is troubling considering faculty of color may need more support based on academic departmental biases in how their scholarly impact is evaluated (Masters-Waage et al., 2024). Therefore, unequal access to material resources may impede faculty of color from producing sufficient research needed for tenure and promotion.

Additionally, racial exclusionary departmental assessment norms have negative implications for faculty of color’s wellbeing. In STEM academic departments, faculty of color explain how they must spend more time and energy to be acknowledged for their scholarly contributions in their STEM academic departments (Blair-Loy and Cech, 2022; Settles et al., 2021). Overworking to be recognized as credible means faculty of color have less time to support their emotional, social, and physical health (Settles et al., 2021). Also, faculty of color explain having to abandon creative scientific projects in favor of those adhering to White Western ways of knowing (Settles et al., 2021). Consequently, these epistemic systemic constraints cause faculty of color to experience less job satisfaction and constraints in academic departments (Settles et al., 2022).

#### 4.2.3 Implications of racialized organizational rule for faculty research production

In summary, STEM academic departments maintain White racial hierarchies through epistemic resource constraints for faculty of color (Dotson, 2014; Settles et al., 2024). STEM academic departments leverage White western norms and values to guide how faculty members are evaluated for their scholarly contributions and potential. By creating epistemic constraints surrounding how research is assessed and valued, STEM academic departments credentialize Whiteness within the organizational promotion structure. Additionally, STEM academic departments decouple from racial equity by not developing organizational actions that address or challenge the epistemic constraints placed on faculty of color. In return, these organizational research rules and routines conducted by STEM academic departments constrain faculty of color’s agency by forcing them to spend exorbitant amounts of time and energy to produce scholarship and status markers deemed worthy of promotion, hindering their wellbeing and retention.

#### 4.2.4 Racialized mechanism in research for students of color

Research participation serves as a training ground for students in STEM academic departments. In research-based universities, faculty research production shapes the amount of funding and status a STEM academic department can receive (Al-Turki and Duffuaa, 2003; Settles et al., 2021). The pressure to produce research creates departmental norms of weighing research higher than other departmental structures like classroom work (DiMaggio and Powell, 1983; Meyer and Rowan, 1977). Students who participate in research are seen as capable of understanding and applying disciplinary concepts in ways that advance the field (Smit, 2010).

The expectation for research participation varies across academic rank in STEM academic departments. At the undergraduate level, students are expected to develop an introductory level skillset like forming research questions, getting familiar with scientific literature, equipment, basic laboratory practices, and sometimes disseminating work to other scientists through publications and conferences. As they move through master’s and doctoral levels, students are expected to become more independent researchers. For example, graduate students are expected to understand how their research sits within their larger field, mentor other students, design and troubleshoot complex experiments, write and publish research manuscripts, and identify and pursue funding opportunities. The successful development of these skills becomes legitimized through the thesis or dissertation process in which graduate students defend a novel research project to a group of experts (e.g., faculty) in their department and university.

Despite the stated prioritization of research, STEM academic departments tend to offer limited opportunity structures for participating in research. STEM academic departments often have informal access structures for research, requiring students to know about the possibility of and actively seek out research opportunities on their own. Additionally, undergraduate students who participate in research often do so through volunteer or unpaid work. Therefore, engaging in research positions in STEM academic departments privileges those who come from wealthier backgrounds or have access to social networks that facilitate gaining access to research opportunities (Davis and Jacobsen, 2014). Students from racially minoritized and economically disenfranchised backgrounds may be forced to pursue paying jobs outside of the university instead of seeking out research positions (Ovink et al., 2024), or may not be aware of research opportunities due to the opacity of the process. Taken together, STEM academic departments not having more centralized research structures that are accessible to all students equally creates additional hurdles for students of color who hold research aspirations.

In STEM academic departments, research lab settings are often spaces entrenched with White racial hierarchies and negative racial climates (NASEM, 2023; Pierszalowski et al., 2021). These climates are maintained through deference rituals and informal interactional norms (Ray, 2019) that define who is a legitimate scientist. Due to these and other already described mechanisms, STEM academic departments are primarily composed of White faculty, especially those on the tenure-track (Baker and Koedel, 2024). Students of color often discuss how professors and peers in research labs enact racial microaggressions or give them ‘service-oriented’ tasks, reflecting cultural schemas devaluing the expertise of non-White populations or lowered expectations of their abilities (McCoy et al., 2017; Morton and Parsons, 2018; NASEM, 2023; Rodriguez et al., 2020).

Additionally, STEM academic departments’ prioritization of White-centered and objective norms negatively impacts the research interests among students of color. Students of color voice how their lived experiences are not always captured in the research opportunities available to them in STEM academic departments (Cech et al., 2017). For instance, scholars like McGee (2021) note how students of color want to conduct research to strengthen their racial and ethnic communities, which is different from White and ostensibly objective ways of knowing in STEM fields (Masta, 2019; McGee and Bentley, 2017; Smith et al., 2014). Tensions between students of color’s interests and White Western ways of knowing may make it hard for students of color to envision STEM as a viable pathway (Cech et al., 2017; McGee and Bentley, 2017; Rodriguez et al., 2019).

#### 4.2.5 Resource constraints in research for students of color

STEM academic departments create racialized and classed disparities in who has access to research opportunities (Ovink et al., 2024). Research participation is strongly correlated with students’ admission into graduate school (Carter et al., 2009; Eagan et al., 2010; Landrum and Clark, 2005), STEM interest and self-efficacy (Linn et al., 2015; Ovink and Veazey, 2011; Strawn and Livelybrooks, 2012), and overall retention (Chang et al., 2011). Relying on Wenger’s (1999) community of practice framing, entry into these research spaces facilitates the essential identity shift from a novice consumer to a legitimate disciplinary participant. Therefore, students of color who participate in undergraduate research can see themselves as producers of knowledge rather than a mere consumer (Carpi et al., 2017; Lane, 2016; Wenger, 1999). However, when informal access structures lock students of color out of these formative communities of practice, it fundamentally undermines their retention and persistence in the field. In other words, the unequal access to resources creates less opportunity for students of color to have access to mentoring networks, research opportunities, and resources that could assist them in gaining skillsets needed to advance their STEM careers.

Additionally, for students of color who can obtain research opportunities, negative racial climates, and research interest constraints can worsen STEM career interest and overall wellbeing (McCoy et al., 2017; Rodriguez et al., 2020). For example, in STEM academic departments, racially chilly climates create pressures for graduate students of color to feel like they must overperform on doctoral milestones to counteract negative cultural stereotypes (Robinson et al., 2016; McGee, 2021; Wilkins-Yel et al., 2019). These racialized expectations may cause graduate students of color to question if they should leave their STEM doctoral programs prematurely (Burt et al., 2018).

#### 4.2.6 Implications of racialized organizational rule for student research production

Collectively, STEM academic departments assist in upholding White racial hierarchies through their organizational routines for student research participation. The decentralized research structure creates unequal research opportunities across racial groups. White students, particularly those who come from educationally and economically privileged backgrounds, are positioned to have greater access to research participation, allowing them to gain more credentials needed to access STEM professions than those who are racially minoritized. Whereas, students of color are denied all those opportunities, as well as the chance to see themselves as knowledge producers, which further affects their retention in the field. Additionally, STEM academic departments’ inability to address the White racial climates and epistemic constraints in their research labs means students of color are forced to navigate these tumultuous environments, creating negative implications for their wellbeing, agency, and future.

### 4.3 Structure 3: instruction

The third structure in STEM academic departments is instruction. Across all colleges and universities, STEM academic departments are called upon to develop curricular structures that will socialize students into disciplinary norms and practices (Ikenberry and Friedman, 1972). Organizational rules require faculty members in STEM academic departments to teach courses, and undergraduate and graduate students are expected to pass these courses. Passing courses reflects a student’s understanding of STEM material and provides students with access to upper-level courses. Students who pass all their coursework are eligible for a degree in their STEM discipline. In other words, instruction is central to the work of universities and academic departments alike.

In this section, we explain how the organizational rules surrounding curriculum and instruction are entrenched with racial inequity. First, we highlight how STEM academic departments’ formal processes for assigning and evaluating teaching often take for granted White and meritocratic assumptions that hinder the agency and resources of racially minoritized populations, particularly faculty of color. As a result, White faculty are better positioned to shape scholarly domains and attend to their wellbeing. Second, while the curricular and instructional structure in STEM academic departments is perceived to work the same for all students, we present counter evidence detailing how this structure hinders and harms students of color in the learning process.

#### 4.3.1 Racialized mechanism in instruction for faculty of color

Teaching labor is organizationally arranged in racially unequal ways in STEM academic departments. For example, in research intensive universities, academic departments allow faculty who bring in research funding to ‘buy out’ their teaching, allowing them to use their time on research rather than teaching (Serow, 2000). Although appearing neutral, buy outs’ can obscure how scientific funding agencies engage in racially exclusionary practices where White faculty receive more funding than faculty of color (Chen et al., 2022; Ginther et al., 2011). These practices potentially preclude faculty of color from benefitting from buy out teaching practices, creating uneven distributions of teaching responsibilities in STEM academic departments. Additionally, teaching large enrollment courses is the primary responsibility for individuals in non-tenure track roles or working at two-year institutions. Systemic inequities in academic department hiring practices contribute to women and faculty of color being overrepresented in contingent faculty positions (Schneider and Bichsel, 2024). Collectively, these racialized organizational arrangements lead to faculty of color being more likely to carry heavier teaching loads than their White counterparts in STEM academic departments. This is particularly troubling in research intensive universities where teaching is often an undervalued part of the promotion and tenure criteria (Rodgers, 2020).

Furthermore, formal teaching evaluations for faculty members are known to be racially exclusionary (McGowan, 2000; Reid, 2010; Stanley, 2006). Although the weight of teaching on promotion, job performance, or tenure varies across colleges and universities, student evaluations are still the most used metric to assess faculty teaching (Shao et al., 2007). Yet, these measures often reflect biases given that research shows that students often hold faculty of color to higher teaching standards than White faculty (Arnold et al., 2016). For example, student evaluations often contain feedback reflecting negative racialized stereotypes toward faculty of color (Reid, 2010). These racial responses are also reflected in how faculty of color report that students undermine their credentials and disrupt their classes in ways not experienced by White faculty (McGowan, 2000; Stanley, 2006). STEM academic departments’ teaching assessments may also overlook how negative racial climates in the classroom environment ensure White faculty have more positive teaching experiences than faculty of color. As mentioned earlier, STEM academic departments have the organizational power to contextualize these evaluations in their promotional assessments for universities and external raters (Misra et al., 2024; Reinholz and Apkarian, 2018). The inability to do so allows for the usage of student evaluations to further create racial inequities in how faculty are retained in STEM academic departments.

#### 4.3.2 Resource constraints in instruction for faculty of color

The uneven assignment of teaching labor hinders the agency and resources of faculty of color in STEM academic departments. First, the rise of academic capitalism has led to the devaluation of teaching and learning (Münch, 2014; Slaughter and Rhoades, 2004). Faculty members whose primary responsibility is teaching or who work in teaching-focused universities receive lower wages than faculty members whose roles or institutions contain other modalities like research (O'Meara et al., 2018). Despite low wages, these faculty members often bear the weight of teaching multiple high enrollment courses each semester and educating hundreds, if not thousands of students per year. The overrepresentation of faculty of color in non-tenure track roles and teaching institutions means they are more likely to receive lower wages than their White counterparts who are more likely to be in tenure-track roles (Schneider and Bichsel, 2024). Additionally, in research universities, faculty of color on the tenure-track, but with high teaching loads have less time to take care of themselves, and attain the high standards for research productivity their institution may expect, hindering their wellbeing and job security (Stanley, 2006).

Also, negative racial climates in college classrooms create additional resource constraints for faculty of color. In classrooms, White racial biases in teaching expectations create environments where faculty of color must develop protective coping mechanisms like code-switching or over-emphasizing their academic credentials to be seen as legitimate knowledge producers by their White students (Harlow, 2003; Pangilinan et al., 2025). And yet, faculty of color explain how these protective coping strategies are not sufficient to eliminate threats of students enacting racial microaggression toward them (O'Meara et al., 2018). As a result, the continued interaction with negative racial climates in the classroom environment has devastating consequences for the emotional and psychological wellbeing of faculty of color in academic departments (Stanley, 2006; Smith et al., 2011).

#### 4.3.3 Implications of racialized organizational rule in instruction for faculty of color

Overall, STEM academic departments uphold White racial hierarchies for faculty through their instructional organizational rules and routines. The teaching arrangements in STEM academic departments via their teaching loads and evaluation metrics contribute to the amplification of agency and resources of White faculty, while constraining the agency and resources of faculty of color. For teaching, the unequal distribution of resources contributes to a lack of retention and promotion among faculty of color, sustaining power in the organizational hierarchy for White faculty.

#### 4.3.4 Racialized mechanism in instruction for students of color

Instruction in STEM academic departments is organized to gatekeep who can pass courses (Sabrowsky, 2023). This gatekeeping occurs through curricular and instructional mechanisms. Curricular mechanisms refer to the organizational actions by which STEM academic departments determine the types of content students should learn to be considered experts in their discipline. Instructional mechanisms refer to the organizational actions surrounding how an individual makes decisions around how content in a course should be taught in a STEM academic department. Together, we argue curricular and instructional structures place racialized constraints on who can pass courses in STEM academic departments.

Curricular mechanisms in STEM academic courses prioritize White ways of knowing and lived experiences (Garriott et al., 2019; Masta, 2019). STEM academic departments determine the curricular standards for student competency (Reinholz and Apkarian, 2018). These standards are informed by disciplinary and university expectations, organizational sites rampant with racial inequity (Dancy et al., 2018; Harding, 2006; Patton, 2016). Based on these influences, the choices STEM academic departments often make around curricular decisions become reflective of White norms and values. First, curriculum in STEM academic departments often reinforces White power structures (Baldo et al., 2005; Hales, 2020; Sahni and Vairaktarakis, 1996). Previously, computer science faculty used language to describe major and minor function features using master/slave analogies (Baldo et al., 2005; Sahni and Vairaktarakis, 1996), although now updated (e.g., manager, worker; Im, 2020). Another example of White superiority in STEM curriculum is reflected in how some biology instructors often struggle with explaining to students the difference between race as a social construction and genetic diversity (Hales, 2020).

Additionally, STEM instruction privileges the experiences of White students (Cech et al., 2017; Garriott et al., 2019; Martin et al., 2013). For instance, some biology textbooks have been shown to credit Darwin for evolutionary advancements, ignoring histories of how Native American tribal communities classified different species (Cech et al., 2017). Further, when STEM educators attempt to create open education resources that center people of color, they inadvertently reify the status quo by positioning communities of color as deficient and in need of help from science and scientists, which can also lead to feelings of White superiority and saviorism in STEM (Couret, 2023).

Instruction in STEM academic departments also reflects racially exclusionary mechanisms. The curricular sequence and assessments of STEM undergraduate majors is organizationally arranged to benefit White students (Fiorini et al., 2023; Hatfield et al., 2022; O’Connor et al., 2015). Structural racism in K-12 educational systems produces unequal advantages in STEM exposure for White students (e.g., academic tracking, racial segregation of schools; Logan and Burdick-Will, 2016; Meatto, 2019). The curricular sequence of STEM college courses often does not address racial and classed inequities in K-12 schooling systems like college preparatory courses and standardized test preparation (Hatfield et al., 2022; Hodara, 2013; Weston et al., 2019). Consequently, the inability of STEM academic departments to have a curricular structure accounting for external racial inequities amplifies institutionalized racism, disadvantaging students of color.

Additionally, introductory ‘weedout’ courses are structured around ensuring large amounts of students cannot matriculate into upper-level STEM courses (Seymour and Hunter, 2019). Across universities, these courses are structurally arranged to make these classes easier to navigate for White students who come from economic and educationally advantaged backgrounds (Battey and Leyva, 2016; Leyva et al., 2021; Park et al., 2021). Even ostensibly equitable instructional practices such as collaborative work in science active learning classrooms, tend to exclude Black students from equitable opportunities to learn due to racial microaggressions and other forms of racial exclusion that faculty fail to perceive (King et al., 2023; Nichols et al., 2026). Further, faculty often teach courses in reductionist ways that do not connect STEM to society or communities, which has been shown to dissuade students of color from pursuing STEM degrees since these traditional ways do not align with their communal goals and equity ethic (McGee, 2021). In addition, students who have access to study groups are more likely to do better in introductory STEM courses (Arendale, 2014; Washington and Mondisa, 2021). Yet, students of color discuss how their White classmates are less likely to include them in peer study groups (Flores et al., 2023; Means et al., 2022; Park et al., 2021), including in class peer groups (Nichols et al., 2026).

#### 4.3.5 Resource constraints in instruction for students of color

Collectively, curricular and instructional mechanisms in STEM academic departments combine to constrain the resources for students of color. Within STEM academic departments, classrooms and office hours are sites where students of color interact with peers and instructors who enact racial microaggressions toward them (Davis et al., 2015; Jack, 2019; McGee and Martin, 2011; Yosso et al., 2009; Tichavakunda, 2021). Exposure to racially hostile curricular environments has devastating effects on the STEM identity development, psychological wellbeing, and access to material resources for women and students of color (Borum and Walker, 2012; Franklin, 2019; Franklin et al., 2014; Martin, 2000; McGee and Martin, 2011; McGee and Stovall, 2015; McGee and Stovall, 2026).

Additionally, White racial hierarchies in STEM academic departments elicit students of color to experience psychological stressors (Steele, 1997). For example, based on White racial hierarchies and related microaggressions and overall racial climate, students of color often experience stereotype threat, a phenomenon when they, consciously or unconsciously, feel at risk of confirming negative stereotypes about their intellectual abilities (Steele, 1997). This can cause anxiety that leads to underperformance in STEM course exams (McGee and Martin, 2011; Rodriguez et al., 2020). Overall, White-centered curriculum and exclusionary instructional practices systematically constrain the psychological and material resources of students of color (Chang et al., 2011; Johnson et al., 2011; Ladson-Billings and Tate, 1995).

#### 4.3.6 Implications of racialized organizational rule in instruction for students of color

STEM academic departments maintain White racial hierarchies among their student population through their instruction. The racialized nature of STEM curricular and instructional processes restricts the agency of students of color by creating racialized disparities in who can pass coursework. These curricular and instructional arrangements further racial disparities in STEM degree conferrals for undergraduate and graduate students. Additionally, students of color who pass coursework, but receive lower grades due to systemic barriers are prevented from being seen as ‘competitive’ for internships, graduate schools, and other professional opportunities. Systematically, the racial gaps in course grades, GPA, and degree conferral rates create less opportunities for students of color to access STEM professions, which also works to reproduce Whiteness as a credential (Hatfield et al., 2022; McGee, 2020; Weston et al., 2019).

### 4.4 Structure 4: service

The fourth structure in STEM academic departments is service. In STEM academic departments, organizational rules require faculty to engage in service responsibilities that support the department and the broader university such as being members of the academic senate, or part of committees on curriculum, research, promotion, hiring, admissions, and awards. In this section, we explain, while the formal rules related to service in STEM academic departments may ostensibly apply equally across all faculty (e.g., a specific ratio of service, teaching, and research expectations), these rules still lead to disparate racialized effects in practice. Additionally, we highlight how STEM academic departments often decouple from racial equity efforts, meaning they are less likely to engage in practices challenging White racial hierarchies and resource allocation (Ray, 2019). In return, the decoupling of STEM academic departments creates informal expectations for faculty and students of color to perform racial equity labor, hindering their agency and resources.

#### 4.4.1 Racialized mechanism in service for faculty and students of color

As discussed, formal rules in STEM academic departments in research intensive universities include valuing research output (e.g., grants and publications) over teaching and service for tenure and promotion. While STEM academic departments in teaching-focused universities and colleges may also value teaching, service tasks often are considered as the least valued and taken for granted type of expected labor in academia. And even within service, some types of tasks may be more valued than others. For example, some service can position faculty in places where they are provided with opportunities for networking and collaboration with senior faculty and administrators. Some practices allow White male faculty to dominate long-standing institutional committees or informal networks and power structures, which exclude faculty of color from networking opportunities that would benefit their careers (NASEM, 2023). Further, White faculty benefit from taking on service assignments that grant them status or at least takes less time and emotional labor (Scott et al., 2024).

In academic departments, faculty of color often take on much of the “invisible labor” that is often informally expected of them but not formally valued by institutions (Arnold et al., 2016). Because they are underrepresented in STEM academic departments (NCSES, 2023), faculty of color are often relied upon by STEM academic departments for providing mentorship, guidance, and support for students of color. Some of this ‘invisible labor’ involves tasks like supporting students of color who are in crisis due to navigating the shifting pressures of institutional demands and the unjust broader sociopolitical context (Diggs et al., 2009; Padilla, 1994). Invisible labor like mentoring the next generation of students of color may be described as important in departmental websites and mission statements, but due to academic departments’ devaluing racial equity work, they are often not formally recognized by the department in terms of merit pay, retention, promotion, and tenure metrics (O'Meara et al., 2018; Stanley, 2006).

Additionally, racialized decoupling practices in STEM academic departments create informal service expectations for students of color (Perez et al., 2022; Nakajima et al., 2022). STEM academic departments’ values of objectivity lead them to decouple from challenging the racialized nature of the department (Blair-Loy and Cech, 2022; Harding, 2006). The minimal challenging done by STEM academic departments is amplified by recent legislation declaring affirmative action and diversity, equity, and inclusion (DEI) policies as discriminatory (Conyers and Wright Fields, 2026). And still, students of color still experience STEM academic departments as racially hostile spaces, hindering them from getting the resources needed to be supported. Thus, students of color often are forced to work toward redistributing academic and career resources back toward themselves (Perez et al., 2022; Nakajima et al., 2022). This racial equity labor often happens via affinity groups and organizations created to support students in specific STEM disciplines (Lerma et al., 2020; Tichavakunda, 2021).

#### 4.4.2 Resource constraints in service for faculty and students of color

In STEM academic departments, formal and informal service rules and norms inequitably distribute resources and opportunities for faculty and students of color. For faculty, service is generally devalued in tenure and promotion decisions. Faculty of color often take on more service that is especially undervalued without additional compensation or reduced teaching and research loads (Stanley, 2006). While most faculty of color appreciate the opportunity to give back to their communities (Guillaume and Apodaca, 2022; Turner et al., 2008), these tasks tend to take more time and emotional labor and can lead to “cultural taxation” (Padilla, 1994). Cultural taxation is the “time consuming” and “emotionally draining” obligation to serve the institution’s “needs for ethnic representation on committees, or to demonstrate knowledge and commitment to a cultural group, which may even bring accolades to the institution, but which is not usually rewarded by the institution on whose behalf the service was performed” (Padilla, 1994, p. 26). For faculty of color, particularly women of color faculty, extra service work leads to differential resource allocation because faculty of color are expected to perform this labor without additional compensation or reduced teaching or research loads (Hirshfield and Joseph, 2012; Rideau, 2021; Stanley, 2006). These unequal expectations also create less time for grant-writing or publishing for faculty of color during their workday (Diggs et al., 2009; Trejo, 2020). White faculty, who may not need or want to engage in this type of service can spend more time focusing on practices that are formally rewarded by the existing departmental structures (Edwards and Ross, 2018). Therefore, faculty of color receive less credit for important, time-consuming, and often emotionally laborious service that affects their ability to complete expectations for promotion and tenure, which disadvantages them in career advancement (Diggs et al., 2009; Stanley, 2006).

Lastly, informal service expectations for students of color also enact racial disparities in STEM academic departments (Nakajima et al., 2022). The racial equity labor students of color perform is often time-consuming, strenuous, and unpaid (Lerma et al., 2020). Consequently, the informal expectations for racial equity labor create less time for students of color to dedicate time toward their courses, engage in co-curricular research opportunities, and support their wellbeing (Perez et al., 2022; Tichavakunda, 2021).

#### 4.4.3 Implications of racialized organizational rule in service for faculty and students of color

STEM academic departments uphold White racial hierarchies through their formal and informal organizational expectations for faculty and students of color to conduct unequal service. Because faculty and students of color carry the weight of service expectations, STEM academic departments can decouple these service responsibilities from White faculty members, while also portraying people of color as “less productive” and therefore not as meritorious as White faculty (Nakajima et al., 2022; Portocarrero, 2023). As a result, service acts performed by faculty and students of color get rendered invisible, while simultaneously normalizing expectations for faculty and students of color to have less resources and agency to support themselves and their academic careers, diminishing their valuable contributions to the department.

## 5 How does the racialized organizational structure of STEM academic departments (re)produce racial inequity?

The impacts of the racialized organization of STEM academic departments in PWIs extend beyond the ivory tower. In this section, we argue the combination of STEM academic departments across predominantly White universities and colleges engaging in racially exclusionary organizational behaviors entrenches White racial dominance and gatekeeping at macro (e.g., state) and individual levels. We describe the processes in which this entrenchment happens. We also explain how the connections between STEM academic departments and macro and individual levels have negative effects for the conditions of racially minoritized people.

### 5.1 Macro-level—How does the racialized organization across STEM academic departments maintain structural racial inequity?

The racialized organization of STEM academic departments in PWIs maintains White racial hierarchies in STEM professions (Ray, 2019). By connecting White cultural schemas to resources, STEM academic departments ensure White racial groups are more likely to receive the resources needed to obtain departmental credentials (e.g., degree, tenure) than individuals who belong to racially minoritized groups. In return, the unequal distribution of STEM credentials across racial and ethnic groups grants White racial groups greater access to STEM professions. Currently, White individuals are overrepresented in STEM academic programs, health care, policy, and industry settings (Fry et al., 2021; NCSES, 2023). Thus, STEM academic departments act as one of the organizing forces surrounding racial stratification in STEM professions in society.

White racial hierarchies across STEM academic departments widens existing racial wealth gaps (Sullivan et al., 2015). The racialized organization of STEM academic departments brings forth racial gaps in STEM degree conferrals. In the United States, STEM degree earners experience greater economic stability than individuals who do not go to college or are non-STEM majors (Kim et al., 2015). Across STEM academic departments, racially exclusionary organizational mechanisms work to sustain lower agency and resources for students of color, hindering their ability to obtain a STEM degree (Budig et al., 2021; Langdon et al., 2011; McGee, 2020). As a result, STEM academic departments’ contribution to racial gaps in STEM degree conferrals worsens racial wealth gaps at the macro level, meaning students of color have less resources to support themselves and their communities and build generational wealth.

Additionally, White racial hierarchies in STEM academic departments sustain White cultural schemas of superiority in STEM professions. Racialized social systems produce societal norms of associating Whiteness with superiority in STEM fields (Bonilla-Silva, 1997; Harding, 2006). The oversaturation of majority populations in STEM professions, created via the racialized organization of STEM academic departments, reifies existing dominant White cultural schemas of superiority. And yet, because STEM academic departments are constructed as neutral and meritocratic organizations, the dominance of White racial groups in STEM professions gets constructed as a byproduct of the intellectual and hard work of White racial groups, and not connected to the active work of STEM academic departments’ racialized organization.

Additionally, White cultural schemas in STEM academic departments aid in the prioritization of White populations in STEM innovation (Dzirasa, 2020; Harding, 2006; Thomas et al., 2021). For example, medical doctors rely on electroencephalography (EEG) procedures to assess a patient’s potential for seizures or tumors (Teplan, 2002). EEG procedures have been shown to incorrectly map the brain activity of non-White peoples with coarse and curly hair textures, due to EEG technology being developed around traditional straight White hair textures (Choy et al., 2022; Etienne et al., 2020). Also, pulse oximeters often struggle with assessing the oxygen levels of individuals with darker skin tones (Fawzy et al., 2022), which has negative ramifications for non-White populations. Collectively, these examples illustrate how the socialization of individuals in STEM academic departments to embrace objectivity ignores how STEM curricula and research privileges White populations at the macro level (Harding, 2006).

### 5.2 Micro-level—How does the racialized organization in a STEM academic department maintain individual racial prejudices?

The racialized organization of STEM academic departments in PWIs maintains individual-level racial animus (Ray, 2019). By racially stratifying the STEM workforce at the macro level, they reify assumptions and biases that people of color do not have the ability or interest in being STEM professionals. Further, as mentioned earlier, STEM academic departments socialize individuals via their research and instructional practices to embrace objective and meritocratic beliefs (Blair-Loy and Cech, 2022; Harding, 2006). The adoption of these beliefs allows individuals in STEM academic departments to overlook and fail to grapple with how STEM academic departments maintain racial gatekeeping via their interpersonal interactions and organizational processes (Blair-Loy and Cech, 2022; Singh, 2026; Russo-Tait, 2022; Russo-Tait, 2023). Consequently, everyday racialized organizational arrangements in STEM academic departments are maintained, allowing for White racial biases to continue among individuals.

Also, White racial hierarchies in STEM academic departments produce negative racial climates between individuals (Ray, 2019). At the individual level, White racial hierarchies in STEM academic departments reify negative racial stereotypes of racially minoritized groups not being credible STEM peoples. In STEM academic departments, students and faculty of color experience interactions with individuals who hold racial prejudices toward their STEM abilities (Flores et al., 2023; Jackson et al., 2023; McGee and Bentley, 2017). These individual-level racial prejudices can impede students and faculty of color from accessing social networks that can assist in their academic and career trajectories. They can also play out in interpersonal interactions such as in microaggressions and lowered expectations described above (Nichols et al., 2026; Russo-Tait, 2022). Additionally, White racial hierarchies in STEM academic departments can spark feelings of isolation, not belonging, and absence of community for students and faculty of color (Burt et al., 2018; Flores et al., 2023; Strayhorn et al., 2013; Settles et al., 2019).

Lastly, the racial organization of STEM academic departments causes racially minoritized populations to experience racial battle fatigue (McGee, 2021; Smith, 2014; Smith et al., 2011). Racial battle fatigue refers to how repeated encounters with systemic racism and racial prejudice negatively affect the emotional, psychological, and physical wellbeing of racially minoritized populations (Smith, 2014). In response to STEM academic departments’ racialized organization, students and faculty of color engage in efforts to redistribute agency and resources back toward themselves and their communities (Nakajima et al., 2022; Padilla, 1994). Although important, the combination of negative racial climates, racial equity labor, and resource constraints prompt students and faculty of color to experience racial battle fatigue (Hartlep and Ball, 2019; Franklin, 2016). In STEM academic departments, students and faculty of color discuss having to spend large amounts of time and energy to overcome racial exclusions in admissions and hiring, research, instruction, and service (Perez et al., 2022; Settles et al., 2019). The exorbitant amount of time and energy required to persevere through these racially exclusionary organizational processes has adverse effects on the physical and mental wellbeing of students and faculty of color (McGee, 2021; Smith, 2014).

## 6 Discussion: How might we repair and reimagine the possibilities of STEM academic departments?

Overall, this manuscript sought to provide a theoretical argument, supported by empirical evidence, about how STEM academic departments in PWIs function as racialized organizations. Current mainstream literature often portrays STEM academic departments as neutral and meritocratic entities, implying their organizational mechanisms work in democratic ways allowing all individuals and knowledge systems equal access and material support (Bess et al., 2023; Harding, 2006; Gonzales et al., 2018). Drawing on Ray (2019), we argue STEM academic departments in PWIs are not true democratic organizations; rather, we demonstrate how the seemingly mundane structures and mechanisms at play in those spaces function to ensure White peoples and knowledge systems are privileged, reinforcing White ideological and material outcomes. We argue this privileging occurs in the form of STEM academic departments valuing White intellectual superiority and other racialized schemas that are coupled to the distribution of resources across racial lines. Through these racialized organizational arrangements, STEM academic departments ensure racially minoritized people and knowledge systems are systematically gatekept from accessing resources needed to survive and thrive.

While we would predict that some of these mechanisms can also occur in STEM academic departments at Minority Serving Institutions (MSIs) and Historically Black Colleges and Universities (HBCUs), this remains an empirical question. McGee (2016) documents how Black and Latino undergraduate STEM majors encounter minimal diversity among STEM faculty and chilly racial climates at MSIs. This work suggests more studies are needed to explore whether and how the routines and mechanisms discussed in the context of PWIs apply equally in STEM academic departments compared to non-PWIs. For example, researchers can ask whether STEM academic departments in those institutions employ distinct cultural schemas or alternative organizational routines that mirror, disrupt, or explicitly contradict the White-centric norms and resource allocation of PWIs. These types of investigations could provide insights for other potential levers for change.

By detailing how racism is embedded in the structures and routines of STEM academic departments, we do not mean to say that their organizational arrangements are fixed or rigid entities. Instead, we argue that STEM academic departments remain as racialized organizations because of the norms and actions of those inside of them. But people can learn to think and do differently—and as such, we suggest that understanding these mechanisms in detail can help us to identify levers for change. That is, STEM academic departments do not have to be captives of their racialized organizational arrangements and have the potential to be altered in racially equitable ways. For example, Collins (2022) notes organizations with exclusionary organizational features can alter their ‘bureaucratic processes toward humanistic ends’ (p. 281). Additionally, McCambly and Colyvas (2023) leverage Ray’s (2019) theory of racialized organizations to argue for ways organizations can abandon White cultural schemas and adopt schemas challenging or dismantling racialization, producing more equitable distributions of organizational resources. Thus, drawing on these perspectives, we argue STEM academic departments in PWIs should invest in ways to dismantle the connection between White cultural schemas and organizational resources.

While we argue that STEM academic departments in PWIs do not currently operate in truly democratic ways, it is important to note faculty governance in most STEM academic departments offers potential for democratic practices. Therefore, change in STEM academic departments has promise because its meso-level positioning allows for grassroots efforts from faculty, staff, and students more easily than at the institutional level. Thus, we call for the emergence of STEM academic departments who will take advantage of this positioning by embracing more equity-oriented frames (McGee and Stovall, 2026). A STEM academic department that leverages equity-oriented frames would allow for all peoples and knowledge systems to be equipped with the agency and resources needed to thrive (McGee and Stovall, 2026). We acknowledge potential solutions for STEM academic departments must be contextualized based on the variation in institutional and geo and sociopolitical locations. However, drawing from existing research on organizational learning and change, we offer some suggestions below of how STEM academic departments could begin their process of becoming more democratic.

### 6.1 Strategies for gatebreaking STEM academic departments

*Engage in political education and reflexivity* on how Eurocentric Western epistemologies and Whiteness manifests in society and STEM academic departments. As attacks on academic freedom and critical scholarship impact teaching and policy across higher education institutions, it is increasingly urgent that we seek and tell the full truth about our academic disciplines and institutions. Specifically, we emphasize the importance of understanding Whiteness because it functions as the unmarked, normative standard against which all practices and people are judged in STEM academic departments. Disrupting Whiteness in STEM academic departments will require STEM leaders and faculty to develop a critical consciousness (i.e., a critical awareness and analysis of injustice; sociopolitical motivation and agency to act; and individual and collective action toward transforming structures; Freire, 1970; Russo-Tait and Rodgers, 2026). To develop readiness for this strategy, we suggest that readers (re)commit to deepened (un)learning. Some low-stakes, high-impact possibilities might be beginning an intergenerational journal club in your department where faculty, staff, and students could come together to read and discuss some of the pieces cited in our reference list.

*Build a critical mass of co-conspirators in departments and beyond* (Love et al., 2021). The old adage, “find your people,” is sage wisdom for achieving success in antiracist change work. Research consistently demonstrates the efficacy of groups of equity-minded institutional actors coming together to tinker with existing systems in ways that bolster systemic change (e.g., Gehrke and Kezar, 2017; McCambly et al., 2025; McNair et al., 2020). The mobilization of people of color and their co-conspirators is key to challenging the existing racial order, but that first demands that we create spaces to gather and build together. Whether it be in the context of professional societies or associations in your discipline, faculty shared governance groups, student advocacy groups, unions, those committed to the fight for racial equity in STEM should consider becoming involved in organizations that support this work (Russo-Tait et al., 2026). Considering the disproportionate burden of racial equity work on people of color, especially those who also identify as women or femmes, we urge White faculty, particularly those who hold resources and status, to provide crucial advocacy and support that enable anti-racist practices to gain an organizational foothold. This could be as small as speaking up in faculty meetings to advocate for change or serving in a leadership position in the department to advance initiatives related that engender the redistribution of departmental resources. As Mariame Kaba reminds us, “everything worth doing is done with other people.” So, find your people and get to work.

*Identify one structure or mechanism in your context to redesign.* Given how deeply entrenched and multi-faceted racial inequity is in academic departments, it is important that we start small. Think of it like a Jenga set—you might not topple the structure by only pulling one block, but strategically pulling at blocks that destabilize the structure will also help topple the structure. Thus, we encourage leaders to identify what is the most urgent and actionable racialized mechanism that, when addressed, is high impact for addressing racialized outcomes. For example, we explained how weed-out courses are structured to privilege White students who have greater access to peer study groups and curricular support, and its curricula also prioritize White ways of knowing and lived experiences. To show if this mechanism is at play in a department, STEM academic departments can examine the DWF rates of individual courses and assess if those grades disproportionality occur among students of color (McNair et al., 2020). Organizational routines, like course design and grading, act as muscle memory that efficiently get work done without much reflection. When these routines reflect Whiteness, they reproduce racial inequality. By slowing down (using an Equity Pause), instructors are forced to confront the gap between the idea of fair grading (ostensive aspect) and the racially disparate practice (performative aspect), allowing them to design new practices that interrupt racialized outcomes.

*Advocacy for change must be directed toward structural solutions.* Bonilla-Silva (1997) urges us to remember that racism is not a moral problem but a systemic one, which demands that we propose solutions that tackle fundamental, root causes to racial stratification. Given that academic departments sit at the “mid-level,” they are a unique lever for change that reverberates to the broader, institutional dynamics as well as micro-level interpersonal discourses and interactions (McGee et al., 2023). Such interventions might include efforts to challenge epistemic oppression in formal evaluation for faculty. For example, STEM faculty could collectively decide to shift departmental promotion and tenure criteria to formally recognize and reward the labor of supporting racial equity, diversity, and mentoring students of color. Such a meso-level intervention would go a long way in mitigating the devaluation of knowledge produced by scholars of color, such as work focusing on social justice issues, disparities, or community-engaged research.

## 7 Conclusion

In this paper, we posited that STEM academic departments in PWIs function as racialized organizations. In other words, we demonstrated how STEM academic departments in PWIs have norms, policies, and practices that systematically connect White cultural schemas to material, economic, and psychological resources, thereby (re)producing White racial dominance in STEM fields (Ray, 2019). The maintenance of this racialized organization often occurs through covert, subtle, and seemingly nonracial dominant practices, coupled with claims of neutrality, objectivity, and meritocracy, validating the White racialized hierarchical structure. By positioning the STEM academic department at the meso-level of analysis, we engage in an essential shift from focusing on individual prejudice to organizational mechanisms (McGee, 2020). Ultimately, we hope our theoretical contribution initiates timely discussions, empirical interventions, and collective actions for making STEM academic departments more democratic organizations. In return, we argue this collective commitment can help contribute to creating racially equitable STEM academic departments where all our racially minoritized students and colleagues have the agency to achieve their goals with dignity, respect, and justice.

## Data Availability

The original contributions presented in the study are included in the article/supplementary material, further inquiries can be directed to the corresponding author.

## Footnotes

1 Although this paper focuses on STEM academic departments, our argument also applies to other academic departmental contexts in PWIs. Racialized organizations do not exist in silos; rather, racialized organizations emerge from being situated in societies with racially oppressive forces (Bonilla-Silva, 1997; Ray, 2019). Therefore, all academic departments in PWIs embedded in racialized societies are likely to engage in racially exclusionary practices to uphold White oppressive forces. However, we focus on STEM academic departments in this paper because of the ways neutral, objective, and apolitical stances are amplified in STEM fields, which work to validate existing racialized organizational arrangements (Blair-Loy and Cech, 2022; Harding, 2006).
